# Laser Synthesis of Iridium Nanospheres for Overall Water Splitting

**DOI:** 10.3390/ma12183028

**Published:** 2019-09-18

**Authors:** Hai-Bin Wang, Jia-Qi Wang, Neli Mintcheva, Min Wang, Shuang Li, Jing Mao, Hui Liu, Cun-Ku Dong, Sergei A. Kulinich, Xi-Wen Du

**Affiliations:** 1 Institute of New-Energy Materials, School of Materials Science and Engineering, Tianjin University, Tianjin 300350, Chinajq_wang@tju.edu.cn (J.-Q.W.); wangmin6029@126.com (M.W.); 15632301425@163.com (S.L.); maojing@tju.edu.cn (J.M.); hui_liu@tju.edu.cn (H.L.); 2 Research Institute of Science and Technology, Tokai University, Hiratsuka, Kanagawa 259-1292, Japan; nnmintcheva@mgu.bg; 3 Department of Chemistry, University of Mining and Geology, Sofia 1700, Bulgaria; 4 Department of Mechanical Engineering, Tokai University, Hiratsuka, Kanagawa 259-1292, Japan

**Keywords:** laser ablated nanoparticles, Ir nanoparticles, bifunctional catalyst, overall water splitting

## Abstract

Engineering surface structure of catalysts is an efficient way towards high catalytic performance. Here, we report on the synthesis of regular iridium nanospheres (Ir NSs), with abundant atomic steps prepared by a laser ablation technique. Atomic steps, consisting of one-atom level covering the surface of such Ir NSs, were observed by aberration-corrected high-angle annular dark-field scanning transmission electron microscopy (HAADF-STEM)**.** The prepared Ir NSs exhibited remarkably enhanced activity both for oxygen evolution reaction (OER) and hydrogen evolution reaction (HER) in acidic medium. As a bifunctional catalyst for overall water splitting, they achieved a cell voltage of 1.535 V @ 10 mA/cm^2^, which is much lower than that of Pt/C-Ir/C couple (1.630 V @ 10 mA/cm^2^).

## 1. Introduction

Electrochemical water splitting has been known as an efficient strategy for the storage of intermittent electrical energy, via hydrogen evolution reaction (HER) and oxygen evolution reaction (OER) [1,2]. The main concern for this technology is to design efficient HER and OER electrocatalysts, especially for acidic media [3,4,5]. As a noble metal, iridium shows a high potential for water splitting [6,7,8,9]. Nevertheless, its performance has barely been satisfactory thus far, due to the inappropriate adsorption of OER and HER intermediates [10,11,12]. Hence, many efforts, e.g., those based on particle size reduction [13,14], use of composites [15,16], and alloying [17,18,19,20], have been made to improve and optimize Ir catalysts. 

Surface structure of catalysts can make a significant impact on the adsorption of intermediates, thus efficiently influencing catalytic properties. For instance, atomic steps on catalyst surfaces were reported to lead to unsaturated catalytic sites [21,22], and exhibit great advantages in CO oxidation [23], ethanol oxidation [24], oxygen reduction [25], and CO_2_ reduction [26]. Nevertheless, common wet-chemistry routes usually give rise to Ir catalysts with flat facets resulted from their equilibrium growth, which severely prevents the formation of atomic steps [20,27,28,29].

Herein, we employed a physical method, pulsed laser ablation in liquid (PLAL) [30,31,32,33,34,35,36,37,38,39,40,41,42] to produce Ir nanospheres (Ir NSs) enriched with surface atomic steps. This method is known as an efficient approach for preparation of various metallic nanoparticles [30,31,40,41,42], including those for electrocatalysis [40,41,42]. The as-prepared catalyst exhibits excellent OER and HER activities superior to those of commercial Ir/C and Pt/C electrodes in acidic medium, indicating that atomic steps on particle surface are advantageous for catalytic performance. After characterization, the as-prepared Ir NSs were employed as a bifunctional catalyst for overall water splitting, achieving a very low cell voltage of 1.535 V @ 10 mA/cm^2^ in 0.5 M H_2_SO_4_, which is about 100 mV lower than that for the Pt/C-Ir/C couple (1.630 V @ 10 mA/cm^2^). Our work proves that PLAL is a promising approach to produce non-conventional surface structures, while the obtained results pave a new way towards highly effective noble-metal catalysts for water splitting.

## 2. Materials and Methods 

**Synthesis of Ir NSs.** Ir NSs were produced by laser ablation of an Ir target immersed in deionized water. Nd:YAG laser (Dawa-350, Beamtech, Beijing, China) was operated at wavelength 1064 nm, pulse width 7 ns, single-pulse energy 250 mJ, and frequency 15 Hz. More details about this preparation approach can be found elsewhere [30,31,32,33,34,35]. More specifically, an Ir plate (30 mm × 30 mm × 3mm, 99.99%) was first polished by sandpaper to remove the surface oxide layer, followed by rinsing with deionized water. Next, the target was immersed in deionized water with its upper surface 20 mm below the water level, and then ablated by the above mentioned pulsed laser for 30 min at room temperature. The as-obtained colloid was mixed with carbon black at a mass ratio Ir/C of 1:4. The mixture was first ultrasonicated and then centrifuged. Finally, the precipitate was dried in a lyophilizer to get the final product, which was used as electrode. 

**Preparation of commercial Ir/C.** The commercial Ir nanomaterial (Hesen, 99.99%) was mixed with carbon black at the same mass ratio, Ir/C = 1:4. The mixture was also first ultrasonicated and then centrifuged. Finally, the precipitate was dried in a lyophilizer to get the final product, which was used as electrode.

**Characterizations of catalysts.** Transmission electron microscopy (TEM) analysis was carried out in an FEI Technai G2 F20 (JEOL, Tokyo, Japan) tool equipped with a field-emission gun, and with energy-dispersive X-ray spectroscopy (EDS) module. High-angle annular dark-field scanning transmission electron microscopy (HAADF-STEM) images were obtained by a JEOL ARM-200F instrument (JEOL, Tokyo, Japan), equipped with a cold-field emission gun and a Cs corrector (CEOS) for probing lenses, which was operated at a voltage of 200 kV. X-ray diffraction (XRD) patterns were measured on a Bruker D8 Advance diffractometer (Bruker, Karlsruhe, Germany) with Cu Kα radiation and a Lynx Eye detector (Bruker, Karlsruhe, Germany). X-ray photoelectron spectroscopy (XPS) analysis was performed using a PHI Quantum 2000 scanning ESCA Microprobe spectrometer (Physical Electronics Company, Austin, TX, USA). Raman spectra were obtained using a DXR Microscope Raman spectrophotometer (Renishaw, London, UK) with laser excitation of 532 nm. 

**Electrochemical measurements.** A CHI660E electrochemical workstation (Chenhua, Shanghai, China) was used for testing the HER and OER performance of different catalysts. For electrochemical tests, 3 mg of catalyst were dispersed into 0.6 mL of deionized water, isopropanol, and 5 wt% Nafion mixture used as solvent (volume ratio: 400 μL:170 μL:30 μL) via sonication to prepare an ink with catalyst. Next, 3.6 μL of the ink was loaded onto carbon fiber paper with a fixed area of 0.3 × 0.3 cm^2^, which was used as the working electrode with a catalyst loading of 0.2 mg/cm^2^. Electrolysis experiments were carried out in a standard three-electrode system with O_2_ (OER)/N_2_ (HER)-saturated 0.5 M H_2_SO_4_. A measurement system was composed of a CHI 600E electrochemistry workstation, working electrode with catalyst loaded on carbon fiber paper, counter electrode (carbon rod), and reference electrode (saturated calomel electrode Hg/HgCl_2_/KCl). The applied potentials were converted with respect to RHE:

$$E_{RHE}\left(V\right)=E_{SCE}+0.242+0.0591\;\times \;pH_{electrolyte}$$

Before recording, the potential of each catalyst was scanned at 50 mV/s between 0 and 1.4 V (vs. RHE) for OER and between 0 and –0.2 V (vs. RHE) for HER, until a stable cyclic voltammogram (CV) was obtained. Afterwards, linear sweeping voltammogram (LSV) curves were recorded at a scan rate of 5 mV/s, and Tafel slopes were obtained by plotting overpotential against log (*J*) from the LSV curves. EIS profiles were recorded under 1.53 V (vs. RHE) for OER in a frequency range from 0.1 to 10^5^ Hz. The electrochemical surface area (ECSA) was measured in the potential window of 1.042–1.142 V (vs. RHE) for OER, using different scan rates of 5, 10, 15, 20, 25, and 30 mV/s. As for the electrochemical measurements of the overall water splitting, we acquired the LSV between 1.0 and 1.8 V at a scan rate of 5 mV/s. All OER LSV curves were corrected for *iR* drop at 95%.

## 3. Results and Discussion

**Figure 1:**
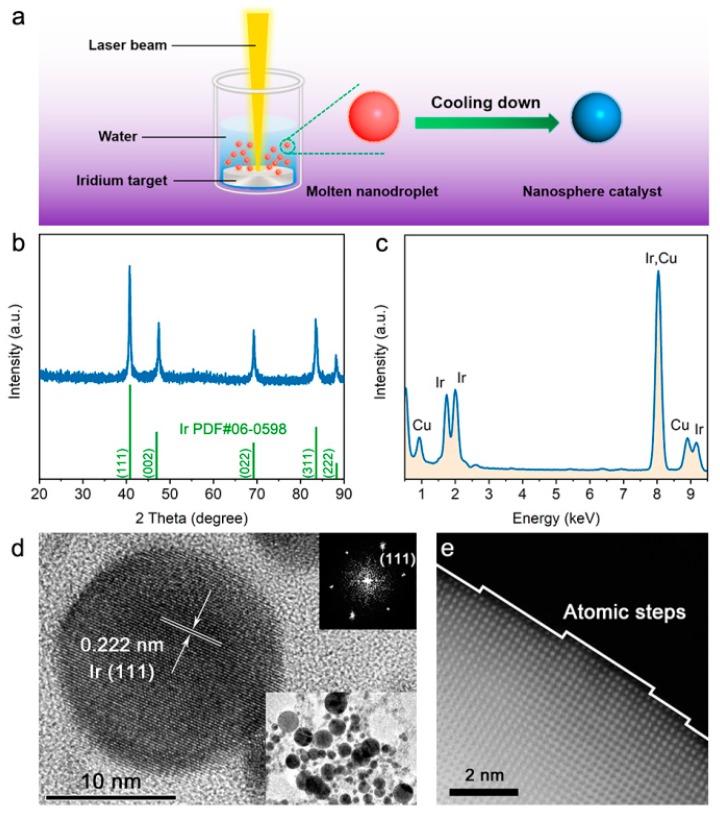
Preparation and characterization of Ir NSs. (**a**) Schematic illustration of preparation procedure involving PLAL. (**b**) XRD pattern and (**c**) EDS spectrum of as-prepared Ir NSs (the Cu signals are from Cu grid used as substrate). (**d**) HRTEM image of a single Ir NS, corresponding FFT (Fast Fourier Transform) pattern and low-magnification TEM image of Ir NSs are given as insets. (**e**) HRHAADF-STEM image of the surface of a single Ir NS.

The preparation of Ir NSs is schematically illustrated in Figure 1a. The Ir metal target immersed in deionized water was ablated by a nanosecond pulsed laser to form vapor and/or molten nanodroplets, which then were quenched by the surrounding liquid medium, resulting in Ir NSs with inerratic cambered surface containing abundant atomic steps. The XRD pattern of as-produced Ir NSs (Figure 1b) shows a typical face-centered cubic structure, indexed as metallic Ir phase (PDF #06-0598). The high purity of Ir nanoparticles is confirmed by the EDS spectrum in Figure 1c and elemental mapping (Figure S1). The high-resolution TEM (HR-TEM) image of a single Ir particle exhibits a regular spherical shape and single crystal structure, with a lattice spacing of 0.222 nm corresponding to the (111) plane of metallic Ir (Figure 1d). The low-magnification TEM image (inset in Figure 1d) reveals that the sample contains Ir NSs with a wide range of sizes from 5 to 40 nm, with an average size being 21.5 nm (Figure S2). More intriguingly, the HAADF-STEM image presented in Figure 1e displays several atomic steps on the particle surface, all with a height of a single atomic layer.

**Figure 2:**
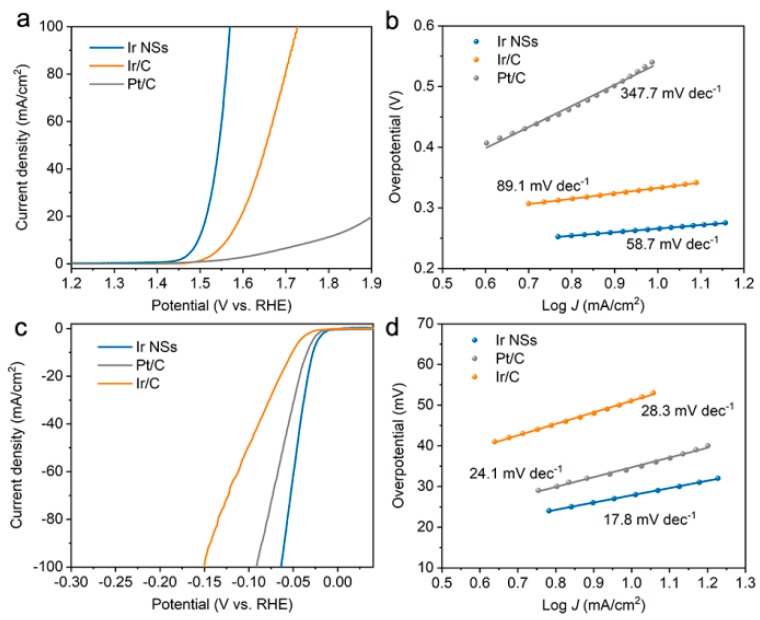
Electrocatalytic OER and HER performance of Ir NSs (blue curves), commercial Ir/C (orange curves), and commercial Pt/C (grey curves). (**a**) OER LSV curves in aqueous solution of 0.5 M H_2_SO_4_ purged with O_2_. (**b**) Tafel plots for OER. (**c**) HER LSV curves in aqueous solution of 0.5 M H_2_SO_4_ purged with N_2_. (**d**) Tafel plots for HER.

Next, upon preparing electrodes, we investigated the OER properties of the Ir NSs in O_2_-saturated 0.5 M H_2_SO_4_ solution, with commercial Ir/C and Pt/C electrodes as references. LSV profiles demonstrated that the Ir NSs exhibited the lowest overpotential of 266 mV to achieve a current density of 10 mA/cm^2^, which is much better than those of the Ir/C (333 mV), Pt/C (547 mV), and other OER electrocatalysts (Figure 2a and Table S1). The Tafel slope of the Ir NSs was determined as 58.7 mV/decade (Figure 2b), which is notably lower than those of the Ir/C (89.1 mV/decade) and Pt/C (347.7 mV/decade) electrodes, indicating the fast kinetics of the laser-prepared catalyst based on Ir NSs. 

**Figure 3:**
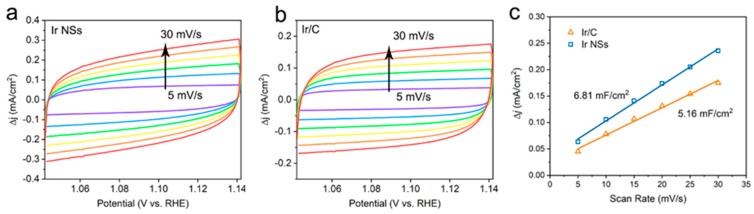
CV curves at different scan rates (5, 10, 15, 20, 25, and 30 mV/s) for (**a**) laser-produced Ir NSs and (**b**) commercial Ir/C in the potential window of 1.042–1.142 V (vs. RHE) for OER. (**c**) Plots of current density versus scan rate to determine double layer capacitance (Cdl) for Ir NSs (blue) and Ir/C (orange) catalysts.

**Figure 4:**
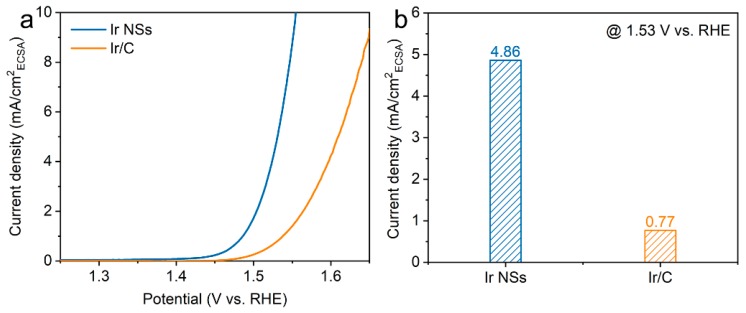
(**a**) ECSA-corrected polarization curves for Ir NSs (blue curve) and Ir/C (orange curve). (**b**) OER current normalized to the ECSA (specific activity) at a potential of 1.53 V (vs. RHE).

**Figure 5:**
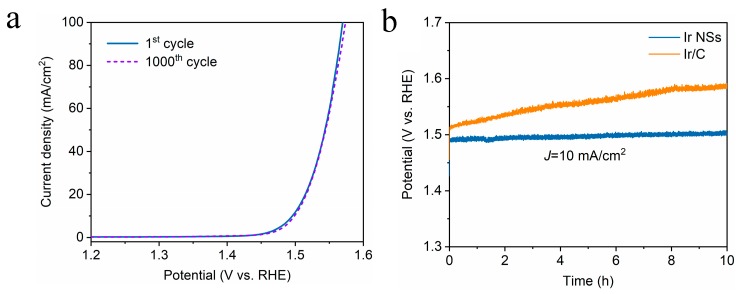
(**a**) LSV curves for Ir NSs before and after 1000 CV scans for OER test in acidic medium. (**b**) Chronopotentiometry curves of Ir NSs (blue) and Ir/C (orange curve) for OER test at a constant current density of 10 mA/cm^2^.

Meanwhile, the ECSA values were determined by integrating the hydrogen adsorption charge on the cyclic voltammogram (CV). As shown in Figure 3c, the value obtained for Ir NSs (6.81 mF/cm^2^) is higher than that of Ir/C (5.16 mF/cm^2^), implying more active sites in the laser-produced Ir NSs in comparison with their commercial counterparts. The intrinsic activities of Ir NSs and Ir/C were evaluated by normalizing the current densities to the ECSA (defined as specific activity), as seen in Figure 4a. The Ir NSs were found to show significantly enhanced specific activity, compared with the commercial Ir/C. At a potential of 1.53 V vs. RHE, the PLAL-generated Ir NSs achieved more than 6-fold improvement in specific activity over commercial Ir/C (see Figure 4b). The results of electrochemical impedance spectroscopy (EIS) presented in Figure S3 show that the charge transfer resistance of laser-produced Ir NSs (69.3 Ω) is significantly lower than that of commercial Ir/C (115.4 Ω), suggesting a faster electron transfer between the Ir NSs and electrolyte. Importantly, the durability of Ir NSs was also found to be very good. As shown in Figure 5a, their OER activity barely changed after 1000 cyclic voltammetry scans. Long-term stability was assessed at a current density of 10 mA/cm^2^, and the electrode based on Ir NSs retained a steady OER overpotential over a period of 10 h, thus being superior to commercial Ir/C (see Figure 5b). In addition, the Ir NSs were found to maintain their spherical morphology after the OER stability test (see Figure S4). 

**Figure 6:**
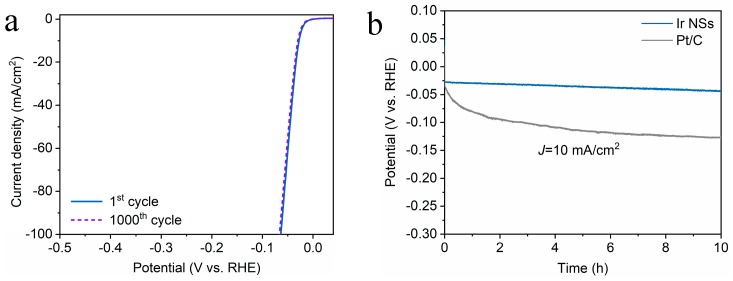
(**a**) LSV curves of Ir NSs before and after 1000 CV scans for HER test under basic conditions. (**b**) Chronopotentiometry curves of Ir NSs (blue) and Pt/C (gray curve) during HER test at a constant current density of 10 mA/cm^2^.

The HER activity of the Ir NSs was examined in 0.5 M H_2_SO_4_ solution purged with N_2_. As shown in Figure 2c,d, the Ir NSs merely need an overpotential of 28 mV to achieve a current density of 10 mA/cm^2^ with a Tafel slope of 17.8 mV/decade, which is significantly lower than similar values for the commercial products Pt/C (34 mV and 24.1 mV/decade) and Ir/C (51 mV and 28.3 mV/decade). Moreover, the laser-prepared Ir NSs exhibit superior durability towards HER (see Figure 6a). During continuous electrolysis for 10 h at a constant current density of 10 mA/cm^2^, they demonstrated a negligible degree of degradation, whereas commercial Pt/C electrode exhibits quite poor stability (see Figure 6b). At the same time, TEM investigations indicated that the morphology of Ir NSs did not change after 10 h of durability testing, thus also confirming good stability of the new catalyst (see Figure S5). 

**Figure 7:**
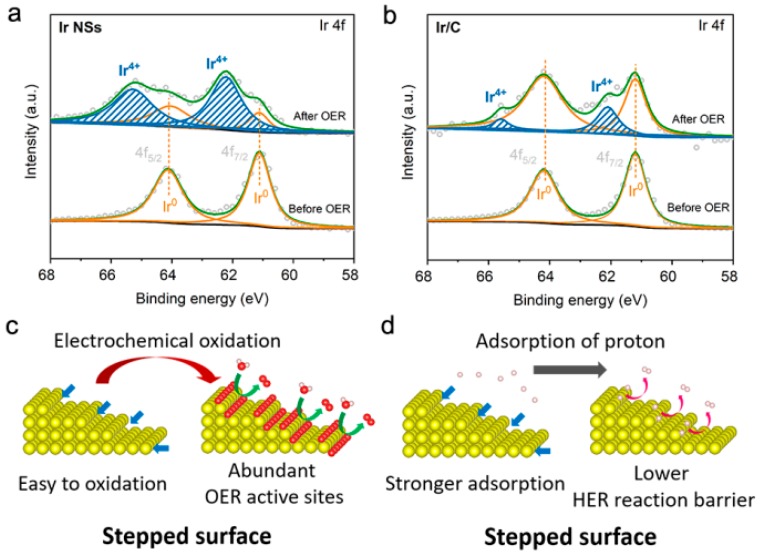
Narrow-scan XPS Ir 4f spectra for (**a**) Ir NSs and (**b**) commercial Ir/C before (bottom) and after (top) the OER test. Proposed mechanisms of (**c**) OER and (**d**) HER on stepped surface of Ir NSs. Blue arrows indicate atomic steps, while green and pink arrows indicate the evolution of oxygen and hydrogen, respectively.

To understand the origin of high performance of the newly developed catalyst based on PLAL-generated Ir NSs, we carried out XPS analysis on both Ir NSs and commercial Ir/C before and after OER tests. The results indicate that after OER test, the Ir NSs were oxidized into IrO_x_ (see Figure 7a), while the nanoparticles in commercial Ir/C product kept their metallic state to a larger degree (see Figure 7b). This finding is further confirmed by Raman results and XPS O 1s spectra (presented in Figures S6 and S7). The observed oxidation of laser-produced Ir NSs can be rationalized as follows. In comparison with the commercial Ir/C particles shown in Figure S8 (with flat facets), the laser-generated NSs are much more chemically active because of numerous atomic steps with lower coordination numbers on their surface [21,22,23,24]. That is why they are easier to be electrochemically oxidized (see Figure 7c and Figure S9a). As well known, electrochemically induced IrO_x_ is much more active for OER compared with metallic Ir [4,12,16], which is why much better OER performance was observed for laser-generated Ir NSs. In addition, the surface steps should also favor the HER activity of the Ir NSs. For metallic Ir catalysts, their weak adsorption energy for H^+^ is known to restrict their HER performance [10,11]. Importantly, the atoms at surface steps possess lower coordination numbers [21,22,23,24], which can improve the adsorption energy of H^+^ and thus reduce the overpotential of HER (see Figure 7d and Figure S9b). Therefore, the high HER performance of PLAL-produced Ir NSs could also be attributed to the plentiful surface atomic steps.

**Figure 8:**
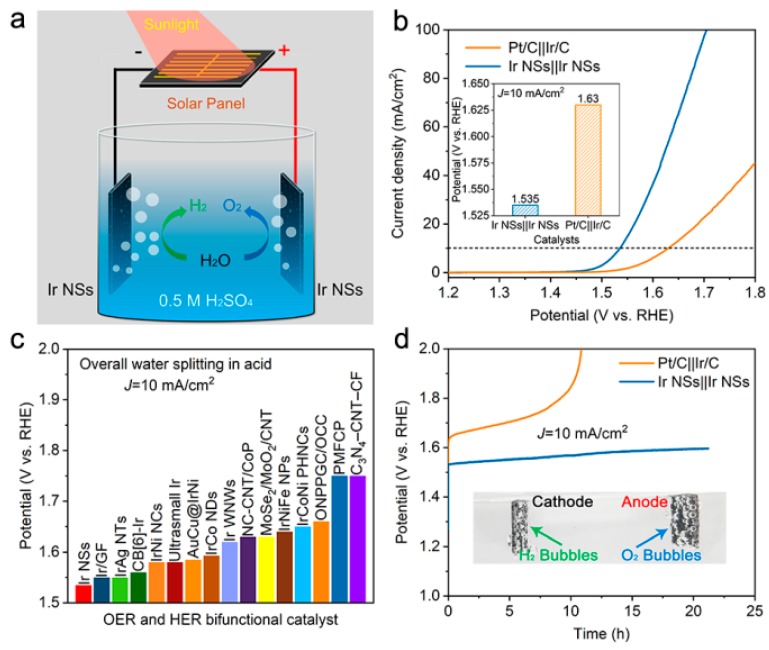
Performance of laser-generated Ir NSs as catalyst for overall water splitting in acidic medium. (**a**) Schematic image of an electrolyzer driven by a solar cell, with Ir NSs as both anode and cathode in 0.5 M H_2_SO_4_ solution. (**b**) LSV curves of Ir NSs and commercial Pt/C-Ir/C couple in aqueous 0.5 M H_2_SO_4_ purged with N_2_ for overall water splitting. (**c**) Comparison of required voltage at a current density of 10 mA/cm^2^ for Ir NSs with other bifunctional catalysts reported in the literature (exact values can be seen in Tables S1–S3). (**d**) Chronopotentiometry test of Ir NSs in comparison with commercial Pt/C-Ir/C couple in aqueous 0.5 M H_2_SO_4_ at a current density of 10 mA/cm^2^. Inset shows an optical image with production of H_2_ and O_2_ on corresponding electrodes.

Given the excellent OER and HER activities demonstrated by the novel catalyst in acidic solution, we employed the Ir NSs as a bifunctional catalyst for overall water splitting in aqueous solution of 0.5 M H_2_SO_4_ (see Figure 8a). As shown in Figure 8b, the Ir NSs exhibited superior activity, with a cell voltage of 1.535 V at a current density of 10 mA/cm^2^. For comparison, the commercial Ir/C-Pt/C couple required a significantly higher cell voltage of 1.630 V to deliver the same current density, which is about 100 mV higher than that for the Ir NSs (Figure 8b). As seen in Figure 8c, the performance of the novel Ir NSs is among the top values achieved by bifunctional catalysts working in acidic solution (also see Tables S1–S3 in supporting information). In addition, the Ir NSs also exhibited very high durability, as the applied voltage for 10 mA/cm^2^ merely increases by 60 mV after 20 h of non-stop operation. This was only 1/6 of that for the commercial Pt/C–Ir/C couple (370 mV after 10 h) (see Figure 8d). The molar ratio of released gases (H_2_ and O_2_) was measured and shown in Figure S10, suggesting the Faraday efficiency of the novel catalyst was nearly 100% at 100 mA/cm^2^. Remarkably, a solar cell with an open-circuit voltage of 1.5 V could drive the water splitting device with obvious and stable gas formation, as well seen in Figure 8d (inset) and Figure S11. Such a solar-power assisted water splitting device can be potentially applied in distributed energy storage technologies.

## 4. Conclusions

In conclusion, using the laser ablation in water, we prepared Ir nanoparticles with numerous atomic steps on their surface. After preparation, the Ir nanoparticles were tested as catalysts for water splitting. The unique surface morphology of the prepared nanoparticles was demonstrated to facilitate their surface oxidation during OER process, and enhance the adsorption of HER intermediate. As a result, the electrode based on the new Ir nanocatalyst demonstrated lower OER and HER overpotentials simultaneously. As a bifunctional catalyst for overall water splitting in acidic medium, the laser-produced Ir nanomaterial provided a current density of 10 mA/cm^2^ at a low voltage of 1.535 V with a long-term stability. The present work demonstrates that laser ablation in liquid phase is a promising technique to prepare metallic nanomaterials with surface atomic steps and improved catalytic performance. This strategy is believed to be capable of preparing other materials and producing novel catalysts for energy conversion and other related applications.

## Supplementary Materials

The following are available online at https://www.mdpi.com/1996-1944/12/18/3028/s1, Figure S1: HAADF-STEM image and the corresponding EDS mapping of Ir NSs showing elemental distribution of Ir (blue), Figure S2: Size distribution of Ir NSs. The average size is 21.5 nm, Figure S3: EIS of Ir NSs and Ir/C recorded at a potential of 1.53 V (vs. RHE), Figure S4: TEM (a) and HRTEM (b) images of the Ir NSs after OER test, Figure S5: TEM (a) and HRTEM (b) images of Ir NSs after HER test, Figure S6: Raman shift spectra of Ir NSs and Ir/C before (a) and after (b) OER test, Figure S7: XPS O 1s spectra of Ir NSs (a) and commercial Ir/C (b) before and after OER test, Figure S8: TEM (a) and HRTEM (b,c) images of commercial Ir/C, Figure S9: Proposed mechanism of OER (a) and HER (b) in flat surface of Ir/C, Figure S10: Faraday efficiency of the corresponding gas products (O_2_ and H_2_) at the current density of 100 mA/cm^2^, Figure S11: Photograph of overall water splitting driven by a 1.5 V solar cell, Table S1: Comparison of OER activity for different electrocatalysts in acidic electrolytes, Table S2: Comparison of HER activity for different electrocatalysts in acidic electrolytes, Table S3: Comparison of overall water splitting activity for different electrocatalysts in acidic electrolytes.
