# Supplementary material for: Laser Synthesis of Iridium Nanospheres for Overall Water Splitting
Source: Materials (Basel). 2019 Sep 18;12(18):3028. doi: 10.3390/ma12183028 (PMC6766323; doi:10.3390/ma12183028)
Supplement: Supplementary file 1 [file materials-12-03028-s001.pdf]

materials

MDPI

Supplementary Materials

Laser Synthesis of Iridium Nanospheres for Overall Water Splitting

Hai-Bin Wang, Jia-Qi Wang, Neli Mintcheva, Min Wang, Shuang Li, Jing Mao, Hui Liu, Cun-Ku Dong, Sergei A. Kulinich and Xi-Wen Du

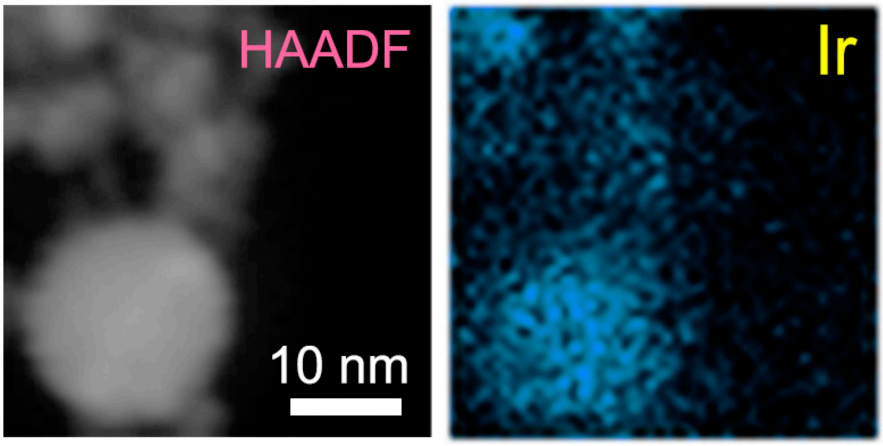

Figure S1. HAADF-STEM image and the corresponding EDS mapping of Ir NSs showing elemental distribution of Ir (blue).

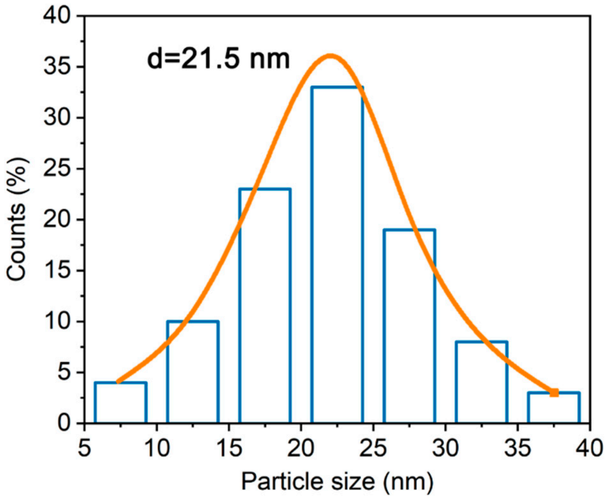

Figure S2. Size distribution of Ir NSs. The average size is 21.5 nm.

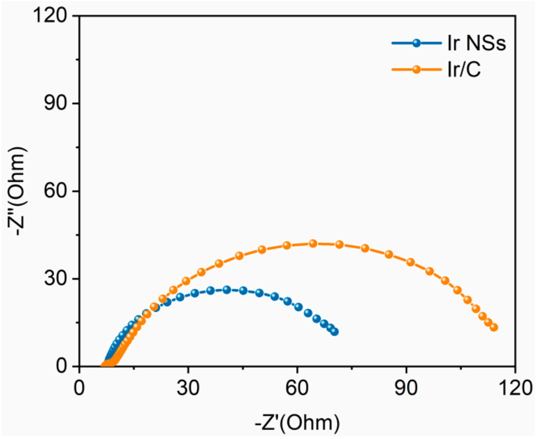

Figure S3. EIS of Ir NSs and Ir/C recorded at a potential of 1.53 V (vs RHE).

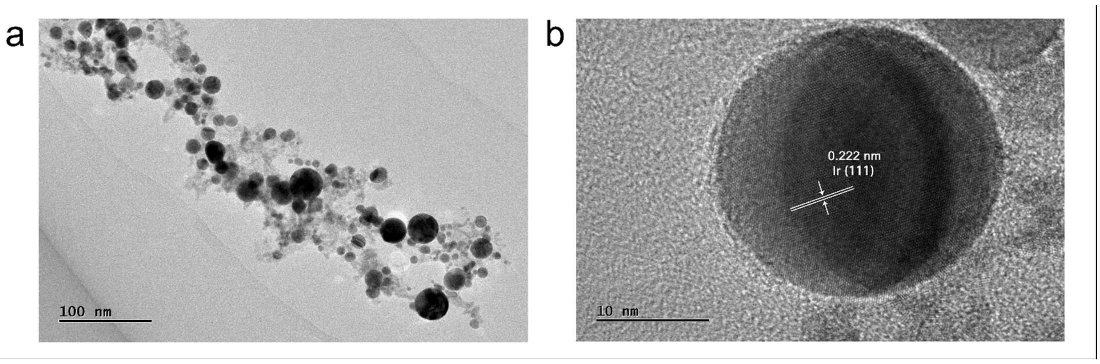

Figure S4. TEM (a) and HRTEM (b) images of the Ir NSs after OER test.

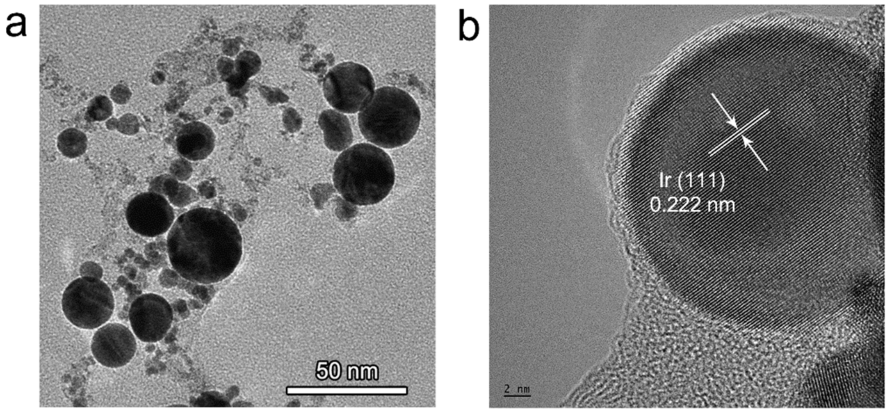

Figure S5. TEM (a) and HRTEM (b) images of Ir NSs after HER test.

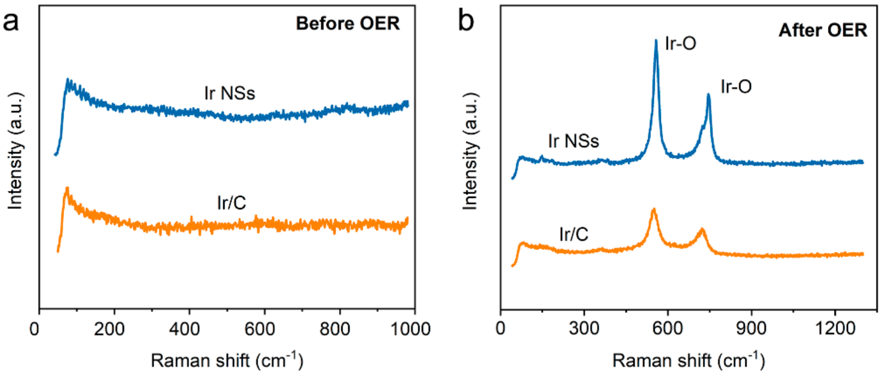

Figure S6. Raman shift spectra of Ir NSs and Ir/C before (a) and after (b) OER test.

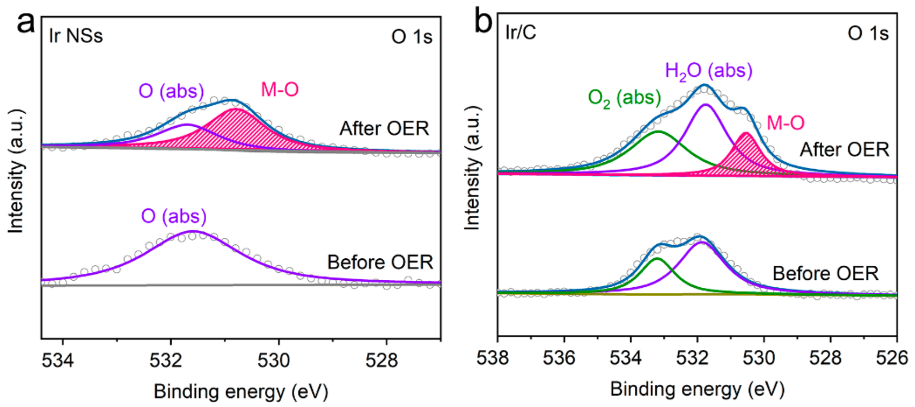

Figure S7. XPS O 1s spectra of Ir NSs (a) and commercial Ir/C (b) before and after OER test.

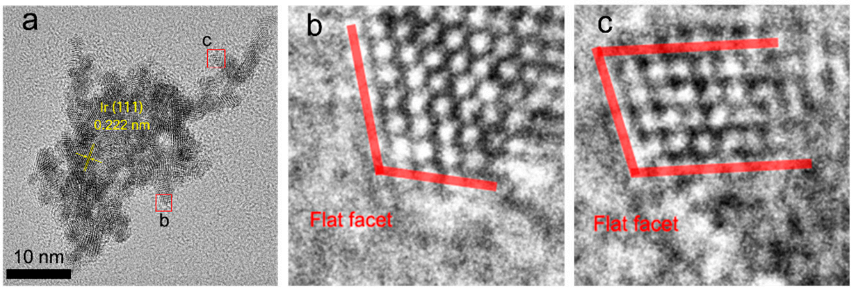

Figure S8. TEM (a) and HRTEM (b, c) images of commercial Ir/C.

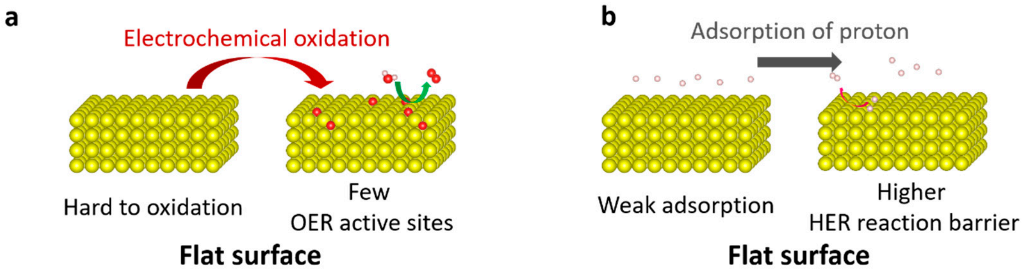

Figure S9. Proposed mechanism of OER (a) and HER (b) in flat surface of Ir/C.

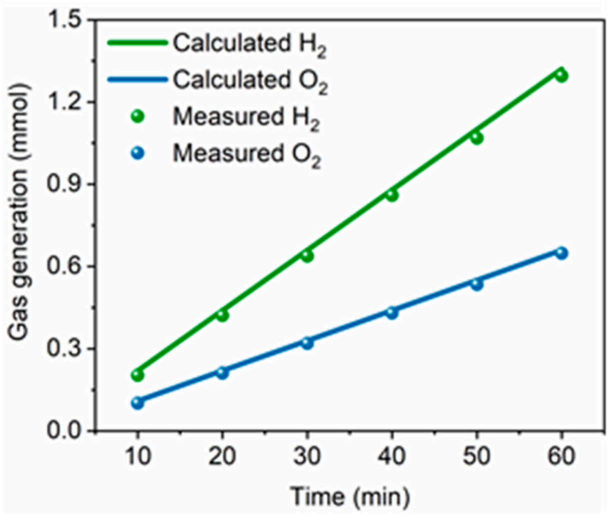

Figure S10. Faraday efficiency of the corresponding gas products (O_2 and H_2) at the current density of 100 mA/cm^2.

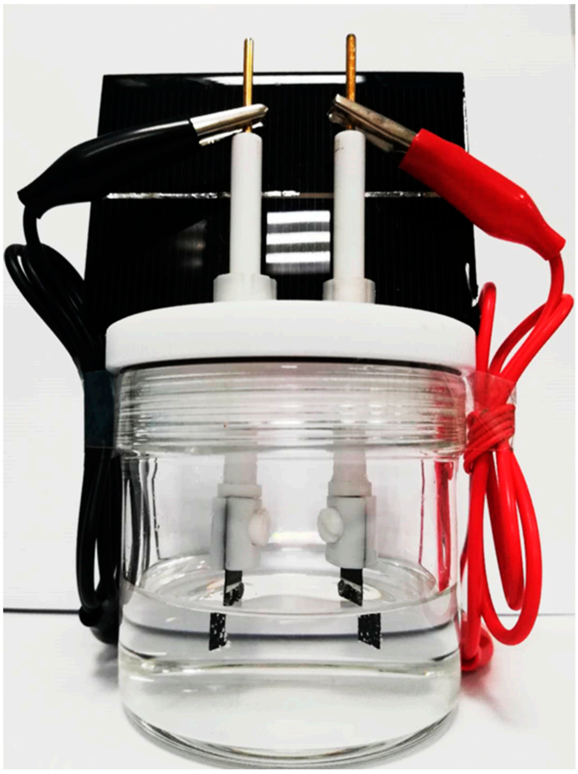

Figure S11. Photograph of overall water splitting driven by a 1.5 V solar cell.

Table S1. Comparison of OER activity for different electrocatalysts in acidic electrolytes.

Catalysts	Electrolyte	Current density	Overpotential	Reference
Ir NSs	0.5M H_2SO_4	10 mA/cm^2	266 mV	This work
Surface-clean 3D Ir	0.5 M $HClO_4$	10 mA/cm^2	303 mV	Nano Lett., 2016, 16, 4424-4430
Ir-Ni NPs	0.05 M H_2SO_4	5 mA/cm^2	348 mV	Chem. Commun., 2016, 52, 5641
IrNi NCs	0.1 M $HClO_4$	10 mA/cm^2	280 mV	Adv. Funct. Mater., 2017, 27, 1700886
Ir-Ni oxide	0.1 M $HClO_4$	10 mA/cm^2	310 mV	J. Am. Chem. Soc., 2015, 137, 13031
Co-IrCu ONC/C	0.1 M $HClO_4$	10 mA/cm^2	293 mV	Adv. Funct. Mater., 2017, 27, 1604688
IrNiCu DNF/C	0.1 M $HClO_4$	10 mA/cm^2	300 mV	ACS Nano, 2017, 11, 5500
$IrNiO_x$/ATO	0.05 M H_2SO_4	10 mA/cm^2	331 mV	Angew. Chem. Int. Ed., 2015, 54, 2975
$IrNi_2$-PE	0.05 M H_2SO_4	10 mA/cm^2	315 mV	ACS Nano, 2018, 12, 7371
Ir_3Cu MAs	0.1 M $HClO_4$	10 mA/cm^2	298 mV	ACS Energy Lett., 2018, 3, 2038
P-$IrCu_x$ NCs	0.05 M H_2SO_4	10 mA/cm^2	311 mV	Chem. Mater., 2018, 30, 8571
Rh_2P	0.5 M H_2SO_4	10 mA/cm^2	510 mV	J. Am. Chem. Soc., 2017, 139, 5494
$IrO_x/SrIrO_3$	0.5 M H_2SO_4	10 mA/cm^2	275 mV	Science, 2016, 353, 1011
IrW	0.1 M $HClO_4$	10 mA/cm^2	300 mV	ACS Central Sci., 2018, 4, 1244

Table S2. Comparison of HER activity for different electrocatalysts in acidic electrolytes.

Catalysts	Electrolyte	Current density	Overpotential	Reference
Ir NSs	0.5M H_2SO_4	10 mA/cm^2	28 mV	This work
IrCoNi-PHNC	0.1 M $HClO_4$	10 mA/cm^2	33 mV	Adv. Mater., 2017, 29, 1703798
Ru@C_2N	0.5 M H_2SO_4	20 mA/cm^2	35 mV	Nat. Nanotechnol., 2017, 12, 441-446
RuP_2@NPC	0.5 M H_2SO_4	10 mA/cm^2	38 mV	Angew. Chem. Int. Ed., 2017, 56, 11559
IrNiN NPs	0.1 M $HClO_4$	6 mA/cm^2	110 mV	J. Mater. Chem. A, 2014, 2, 591
Ru/C_3N_4/C	0.5 M H_2SO_4	10 mA/cm^2	70 mV	J. Am. Chem. Soc., 2016, 138, 16174
Pt_3Ni_3 NWs	0.5 M H_2SO_4	10 mA/cm^2	30 mV	Angew. Chem., 2016, 128, 13051
N-WC	0.5 M H_2SO_4	10 mA/cm^2	113 mV	Nat. Commun., 2018, 9, 924
Co-MoS_2	0.5 M H_2SO_4	10 mA/cm^2	60 mV	Chem. Commun., 2018, 54, 3859
Rh/Si	0.5 M H_2SO_4	50 mA/cm^2	110 mV	Nat. Commun., 2016, 7, 12272
Rh-MoS_2	0.5 M H_2SO_4	10 mA/cm^2	47 mV	Adv. Funct. Mater., 2017, 27, 1700359
Mn-doped CoS_2	0.5 M H_2SO_4	10 mA/cm^2	43 mV	ACS Energy Lett., 2018, 3, 779
PtFeCo	0.5 M H_2SO_4	10 mA/cm^2	50 mV	Adv. Mater. 2016, 28, 2077
Rh_2S_3	0.1 M $HClO_4$	10 mA/cm^2	117 mV	Energy Environ. Sci. 2016, 9, 850

Table S3. Comparison of overall water splitting activity for different electrocatalysts in acidic electrolytes.

Catalysts	Electrolyte	Current density	Potential	Reference
Ir NSs	0.5M H_2SO_4	10 mA/cm^2	1.535 V	This work
Ir/GF	0.5 M H_2SO_4	10 mA/cm^2	1.55 V	Nano Energy, 2017, 40, 27
IrAg NT	0.5 M H_2SO_4	10 mA/cm^2	1.55 V	Nano Energy, 2019, 56, 330
CB[6]-Ir	0.5 M H_2SO_4	10 mA/cm^2	1.56 V	ACS Energy Lett., 2019, 4, 1301
IrNi NCs	0.5 M H_2SO_4	10 mA/cm^2	1.58 V	Adv. Funct. Mater., 2017, 27, 1700886
Ultrasmall Ir	0.5 M $HClO_4$	10 mA/cm^2	1.58 V	Inorg. Chem. Front., 2018, 5, 1121
AuCu@IrNi	0.5 M H_2SO_4	10 mA/cm^2	1.585 V	Nanoscale Horiz., 2019, 4, 727
IrCo NDs	0.1 M $HClO_4$	10 mA/cm^2	1.593 V	ACS Appl. Mater. Interfaces, 2018, 10, 24993

Ir WNWs	0.1 M $HClO_4$	10 mA/cm^2	1.62 V	Nanoscale, 2018, 10, 1892
NC-CNT/CoP	0.5 M H_2SO_4	10 mA/cm^2	1.63 V	J. Mater. Chem. A, 2018, 6, 9009
$MoSe_2/MoO_2$/CNT	0.5 M H_2SO_4	10 mA/cm^2	1.63 V	Nanoscale, 2018, 10, 9268
IrNiFe NPs	0.5 M $HClO_4$	10 mA/cm^2	1.64 V	J. Mater. Chem. A, 2017, 5, 24836
IrCoNi PHNCs	0.5 M H_2SO_4	10 mA/cm^2	1.65 V	Adv. Mater., 2017, 29, 1703798
ONPPGC/OCC	0.5 M H_2SO_4	10 mA/cm^2	1.66 V	Energy Environ. Sci., 2016, 9, 1210
PMFCP	0.5 M H_2SO_4	10 mA/cm^2	1.75 V	ChemSusChem, 2019, 12, 1334
C_3N_4–CNT–CF	0.5 M H_2SO_4	10 mA/cm^2	1.75 V	J. Mater. Chem. A, 2016, 4, 12878
